# Enacting policy to use administrative data for surveillance of cardiac implantable electronic device infections in Alberta, Canada

**DOI:** 10.1017/ash.2026.10874

**Published:** 2026-10-01

**Authors:** Elissa Rennert-May, Derek Chew, Madelyn Knaub, Andrew Frank-Wilson, Stephen Wilton, John Conly, Jennifer Ellison, Stephanie Smith, Kathryn Bush, Evan Martow, Dustin Johnson, Claude Oppenheim, Jenine Leal

**Affiliations:** 1 Department of Medicine, University of Calgary, Calgary, Canada; 2 Snyder Institute for Chronic Diseases, University of Calgary, Calgary, Canada; 3 O’Brien Institute for Public Health, University of Calgary, Calgary, Canada; 4 Microbiology, Immunology and Infectious Diseases, University of Calgary, Calgary, Canada; 5 Department of Community Health Sciences, University of Calgary, Calgary, Canada; 6 Libin Cardiovascular Institute, University of Calgary, Calgary, Canada; 7 Department of Cardiac Sciences, University of Calgary, Calgary, Canada; 8 Infection Prevention and Control, Alberta Health Services, Calgary, Canada; 9 Department of Medicine, University of Alberta, Edmonton, Canada; 10 Infection Prevention and Control, Interior Health Authority, British Columbia, Canada; 11 Division of Cardiology, University of Alberta, Edmonton, Canada; 12 Division of Cardiology, Chinook Regional Hospital, Lethbridge, Canada; 13 Patient Partner, University of Calgary, Calgary, Canada

## Abstract

**Objective:**

Cardiac implantable electronic device (CIED) implantations are common and expensive. Tracking post-surgical infections of these devices is a key component of monitoring health system performance and ensuring high quality care. Given human resource constraints with traditional infection prevention and control (IPC) programs, CIED infections cannot be systemically monitored in many jurisdictions. This project endeavored to implement a policy change in Alberta, Canada, using administrative data for tracking CIED infection trends over time.

**Methods:**

Prior work validated the use of administrative data to track CIED infections. The current work focused on exploring policy options to optimize this use of administrative data and elicit feedback from knowledge users/operational leaders via a workshop and electronic communications. Options for presentation and communication modalities of data including quarterly reports or Tableau dashboards were explored.

**Results:**

Based on consensus from the workshop and subsequent electronic communications, three policy options were provided along with a recommended choice as well as preferred reporting options. The final options were all included in a policy brief provided to operational leadership. Ultimately, the recommended approaches of using administrative data for surveillance were accepted resulting in a policy change for CIED infection surveillance in Alberta that is currently being implemented and evaluated.

**Conclusions:**

CIED infections are a devastating complication following implantation. Administrative data may offer an alternative to traditional IPC surveillance given constrained resources. Engagement and presentation of evidence-based policy options resulted in the adoption of a novel data driven policy in Alberta for CIED infection surveillance.

## Introduction

Cardiac implantable electronic device (CIED) infections are a major complication of CIED surgery, occurring in approximately 1%–5% of patients with these devices, and rising at a rate that surpasses the growth in CIED implantation.^1,2^ This disproportionate rise likely reflects the expanding use of CIEDs among older, more comorbid patients, a population inherently more vulnerable to procedural complications.^3^ CIED infections are life-threatening, often challenging to both diagnose and manage due to their clinical complexity. Patients who develop a CIED infection experience nearly a three-fold higher risk of mortality compared with those who remain infection-free.^2,4^

The increasing rate of CIED infections and complexity of management has been recognized by the American Heart Association as a “health crisis.”^5^ However, there remains a relative lack of wide-spread surveillance programs to monitor for CIED infections. Infection prevention and control (IPC) surveillance programs for postoperative infections are a key component of health system quality assurance.^6^ Surveillance has been identified as a necessary component of any IPC program which can be used to implement strategies to reduce infection risk.^7^ However, these programs can be resource-intensive requiring personnel, infrastructure and substantial funding.^8^

Surveillance utilizing administrative data may offer a cost-efficient alternative given fewer human resource requirements, and the potential for automated algorithmic CIED infection identification. Prior work has been completed validating algorithms using International Classification of Diseases 10^th^ Revision (ICD-10) codes and Canadian Classification of Health Intervention (CCI) codes to identify complex (ie, deep/organ-space) CIED infections.^9^ These algorithms had sensitivity and specificity of over 90% against a gold standard cohort of manually identified CIED infections through chart review and case discussion.^9^ While comprehensive IPC surveillance remains the gold standard for identifying postsurgical infections,^10^ given the personnel requirements, it is not practical for this to be used at every site for each postsurgical infection.

The current work uses the potential of administrative data to synthesize and provide assessment of current and historical trends of CIED infections in the province of Alberta, Canada to inform different policy options for adaptation, and guide change/facilitate processes within IPC surveillance programs and structures. CIED infections were selected as the focus for this policy initiative for three reasons. First, foundational validation work establishing a high-performing administrative data algorithm already existed for this population, providing a ready evidence base for policy translation.^9^ Second, CIED infections carry a disproportionate mortality and cost burden relative to their frequency.^2,4,11^ Third, CIED implantation in Alberta occurs at a small, discrete number of centers, making provincewide administrative surveillance logistically feasible as an initial proof-of-concept for this approach. This work may serve as a template that could subsequently be extended to other implant-related surgical infections (eg, orthopedic, neurosurgical) pending similar validation in those populations.

## Methods

### Overview

This was a policy-focused study evaluating different options for using administrative data to support IPC surveillance of CIED infections. Our objectives were to outline feasible surveillance approaches, engage with key knowledge users to review and refine these options in an iterative fashion, and ultimately inform the operationalization of a provincewide policy change. The study was conducted in Alberta, Canada (population ∼5 million), where CIED implantations occur at six centers and no standardized provincial surveillance system for CIED infections existed. At the time of the study, only one center conducted comprehensive manual surveillance, highlighting substantial variability in site-based practice and the need for a unified provincial approach.

Extensive prior foundational work was done to validate the use of administrative data for CIED infections,^9^ explore the epidemiology of these infections^12^ and look at the economic burden associated with them.^11^ CIED infections were defined as complex (deep or organ-space) surgical site infections meeting Centers for Disease Control/National Healthcare Safety Network criteria—that is, infections involving the device pocket, leads, or associated bloodstream/endovascular structures and typically requiring device and lead removal. Superficial incisional infections not requiring system extraction were not included. This definition, and the ICD-10 codes used to operationalize it, were established and validated against a chart-review gold standard in prior work.^9^ The ICD-10 codes included were: infection of an implantable cardiovascular or other device (T827, T857), infective endocarditis (I330, I339, I38, I398), or cellulitis of the chest wall or other unspecified site (L0330, L0339, L038, L039).

All of these findings were compiled into a summary and presented to knowledge users across the province at a virtual recorded workshop via Zoom (video conferencing software) including physicians who implant CIEDs, infectious diseases physicians who manage these infections and IPC staff and leadership who develop and implement provincial surveillance methods and standards, as well as a patient partner. This process ensured provincewide representation. Additionally, reporting options (ie, Tableau dashboard vs quarterly paper reports vs using both together) were presented followed by targeted questions and leading up to an open discussion and feedback from all participants. A follow up questionnaire was sent to all members to elicit any further feedback and allow knowledge users who were unable to attend the workshop an opportunity to provide comments (see Supplemental Material 1 for the targeted questions that were included in the workshop and follow-up email). All responses gathered from the questionnaire were compiled in a spreadsheet in Excel (Microsoft Corporation, 2025, Version 16, Richmond, WA).

An automated mechanism for extracting administrative data for both the denominator (ie, CIED implantations) and numerator (CIED infections) and presenting it in a Tableau dashboard (Tableau Software, Seattle, WA) was explored to create a hypothetical version of how a dashboard could appear.

### Data sources

Hospital-based health care in Alberta is organized into a province wide single health system that serves the province of Alberta, Canada. The Alberta Health Services (AHS) Data and Analytics department provides integrated access to the Alberta subset of the Canadian Institute of Health Information’s (CIHI) Discharge Abstract Database (DAD), CIHI’s National Ambulatory Care Reporting System (NACRS), Alberta Health’s Vital Statistics—Death Registry, and the Alberta Health Care Insurance Plan Registry through AHS’ Enterprise Data Warehouse. These integrated data sources contain information on all Alberta residents with an Alberta Health Care Insurance Plan (>99% of provincial coverage).

During the period from January 1, 2023, to December 31, 2023, we utilized CCI codes from AHS Analytics DAD and NACRS to identify both the total number of *de novo* CIED implantation and revision surgeries as separate categories. The algorithm operates on linked administrative records as follows: (a) the CIED implantation date is identified as the admission/implantation date associated with a qualifying CCI procedure code for CIED implantation or revision; (b) an infection diagnosis is identified as the presence of one of the specified ICD-10 codes listed above on any subsequent hospital admission or emergency/ambulatory care record for that patient; and (c) the 90-day window is calculated as the interval between the index CCI implantation date and the admission date associated with a qualifying infection code, with only infections occurring within this window counted as CIED-related.

### Analyses

Descriptive statistics were used to create a report prototype and populate the potential dashboard including rate of infection and the number of days and number of procedures completed since last infection, stratified by site of CIED implantation. However, the intent of this work was to explore how a dashboard and/or report could be created to use administrative data for CIED infection surveillance and potentially inform policy change, rather than look at rates of infection which has been explored previously.^12^ Workshop recording notes and free-text questionnaire responses were analyzed using the Framework Method,^13^ a qualitative approach suited to applied, multi-disciplinary health policy research. Three members of the study team jointly familiarized themselves with the workshop transcript and questionnaire responses, developed a working set of themes related to the feasibility, acceptability, and reporting preferences associated with each policy option, and jointly indexed and charted the data from both sources against this framework. Consensus was defined as convergence of opinion across stakeholder groups, with no unresolved substantive disagreement after this iterative charting and discussion process. The final recommended approach was arrived at through this consensus-building process rather than a formal vote.

## Results

### Key findings from knowledge user engagement

There were 29 knowledge users who were invited to attend the workshop and/or respond to the follow up email. These knowledge users represented implanting physicians and Infectious Diseases physicians from across Alberta, and IPC professionals and members of the IPC provincial surveillance teams, as well as a patient partner. Of the 29 invited knowledge users, 17 attended the live workshop and three responded to the follow up email (69% response rate in total). This was made up of five implanting physicians, five Infectious Diseases physicians, one patient partner, 6 IPC professionals/members of provincial surveillance, and three operational leaders.

**Figure 1.**
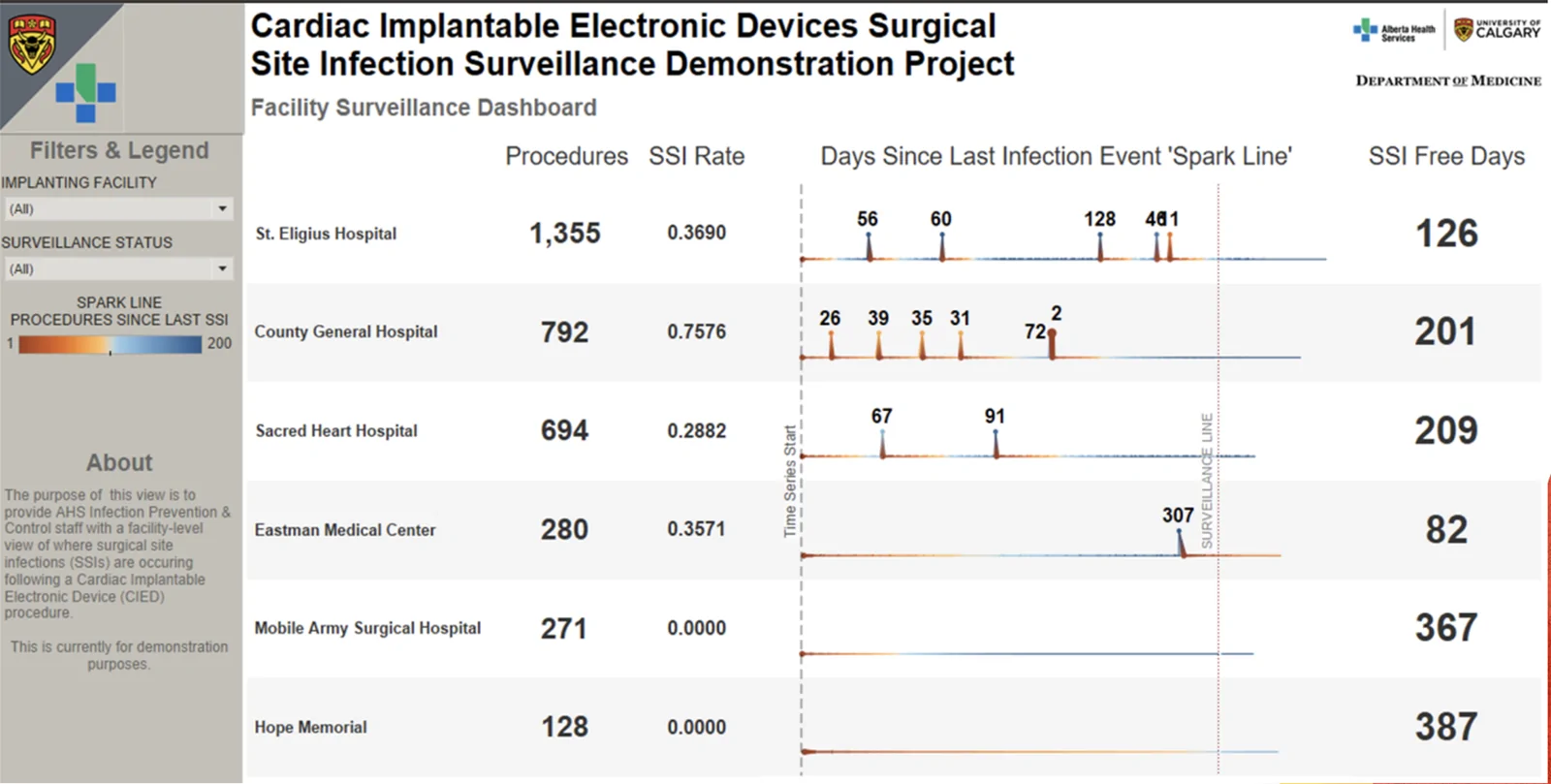
Figure 1 long description. Prototype of dashboard for surveillance data (illustrative example).

To demonstrate the reporting concept to knowledge users, a prototype dashboard populated with illustrative (non-identifying) values was created (Figure 1), showing rate of infection and days/procedures since last infection stratified by implanting site. This prototype was used to elicit feedback on format and content and does not represent confirmed provincial infection rates, which have been reported separately.^12^

We confirmed that using administrative data to track CIED infections has the potential to address current gaps in infection surveillance in Alberta. Key knowledge users expressed interest and excitement with this approach to surveillance. Through these conversations, positive and negative attributes of this work were identified collectively.

### Policy options

The following policy options were created after the workshop and presented as a briefing note to the provincial IPC surveillance and standards team to help inform decisions on implementing CIED infection surveillance (see Supplemental Material 2 for the briefing note including positive and negative attributes of all reporting and policy options).

1. No change in current CIED surveillance practices. Administrative data monitoring is not an acceptable alternative.
2. Use the administrative data to monitor trends for the entire province. If trends change, this triggers more intensive IPC surveillance (**recommended approach**).

This option is likely cost-effective, provides all sites within the province with some type of surveillance, and allows for case verification through comprehensive IPC surveillance.

1. Only use the administrative data for CIED surveillance for entire province and discontinue any traditional IPC surveillance (alternative). Discontinuation of any established reporting or exchange between local IPC surveillance and cardiac program personnel and potentially remove the opportunity for conjoint detailed case-based error reduction rounds or patient safety reviews.

The recommended approach (Policy Option 2) was selected by consensus following iterative discussion among knowledge users at the workshop and through follow-up questionnaire responses, rather than by formal vote, using the methodology described above.^13^

### Policy decision

Based on the evidence and policy options provided, the provincial IPC Surveillance and Standards program in Alberta is adapting this work to trial utilizing administrative data for CIED infection surveillance using policy option 2. They will be following all adult patients in Alberta with CIED implantation for 90 days from index date of implantation and both standard written reports and a dashboard will be created to provide the findings and trends from this surveillance. To assess the impact of this policy as a novel application of administrative data and mechanism for IPC surveillance, on an annual basis cases identified using administrative data will be sent to Infection Control Professionals for review and confirmation of a complex infection. This will also ensure the accuracy of coding and may improve it longitudinally. Any sites that were previously completing traditional IPC surveillance would continue to do this as well. A prespecified statistical threshold for triggering traditional IPC investigation (eg, a defined percentage increase over a rolling baseline rate, or a signal derived from statistical process control charts) has not yet been formally established and represents a critical next step as this policy is operationalized. Development and validation of this trigger threshold, informed by baseline provincial infection rates, is planned as part of the ongoing implementation and evaluation of this policy.

## Discussion

Comprehensive manual IPC surveillance is “gold standard” for infections after CIED implantation and is considered a core component of many IPC programs^7^ however it is human resource intensive and costly. When it is not possible to do this level of surveillance, administrative data may offer a novel alternative. A provincial IPC committee in Alberta will now adapt this and trial it as a mechanism of province wide surveillance demonstrating the successful operationalization of a policy change for IPC surveillance.

The American Heart Association has a national CIED infection initiative aimed at addressing gaps in awareness of detection and treatment of CIED infections.^5^ This initiative includes a national CIED infection summit and a national education plan to provide guidelines, educational resources and care pathways.^5^ Using administrative data as a mechanism for IPC surveillance can complement these other initiatives to reduce CIED infections through timely identification of trends in CIED infections allowing for investigation and intervention when necessary.

This policy change has several strengths. Firstly, this type of surveillance can largely be automated, thus reducing human resource requirements and cost. Exploring ways to adopt this type of policy change is necessary given that as health systems continue to enhance their use of automation and artificial intelligence, including in IPC,^14^ there will be increased need for collaboration and processes to support change. This policy will improve health equity as it can be available at all sites offering CIED implantation even if there are not the resources for wide scale manual CIED infection surveillance on an ongoing basis. This type of work can also be used as a blueprint for other types of infection surveillance (eg, neurosurgery, cardiac surgery, orthopedic surgery) where comprehensive traditional surveillance is not possible due to limited human resources. This may lead to enhanced collaboration between IPC and different surgical services and offer opportunities for patient safety reviews and improvements in patient outcomes.

This work is limited in that prior to this being adapted to other types of surgery there would need to be a comprehensive robust evaluation and validation work done on the administrative data to ensure accuracy in the codes at identifying infection. Additionally, validation work should be done in alternative regions outside of Alberta to ensure the reliability and generalizability. Unlike jurisdictions with diagnosis-linked reimbursement, Alberta hospitals are not paid per diagnosis code; rather, coded discharge abstracts (including diagnosis and procedure codes) are a mandatory component of provincial and national hospital reporting creating a structural incentive for complete and standardized coding independent of any direct reimbursement link. In jurisdictions where standardized diagnostic coding is not similarly mandated or systematically audited (eg, systems relying on optional or fee-for-service-only coding) the sensitivity and completeness of an administrative-data algorithm such as this one may be reduced, and local validation would be needed before adoption. This model relies on provincewide linkage of administrative data across a single, centralized health system covering >99% of residents, which is not universally available. In more fragmented healthcare systems (eg, those with multiple competing payers, non-interoperable electronic health records, or incomplete hospital-level reporting) the completeness of the denominator (CIED implantations) and numerator (infection-coded encounters) would need to be assessed.

Another potential limitation of this approach is the inherent latency of administrative data. CIED implantation and infection-related hospital encounters typically become available in the administrative data extracts only after a reporting lag of approximately 12 weeks. However, as traditional IPC surveillance is typically not reported more than quarterly this is not anticipated to create a substantial time delay. Finally, this type of surveillance is useful largely for monitoring trends as infection rates will be overestimated due to their uncommon occurrence. While the ICD-10 algorithm used in this work has excellent sensitivity (90.6%) and specificity (98.6%) against a chart-review gold standard, its positive predictive value is considerably lower (32.5%), reflecting the low baseline prevalence of CIED infection.^9^ In practical terms, this means that when the algorithm flags a case as a possible complex CIED infection, it will be a true infection roughly one-third of the time, and IPC teams reviewing flagged cases for confirmation should expect a corresponding chart-review workload of approximately two false positives for every confirmed true positive (given overall low infection rates this is not anticipated to be a substantial workload burden). This is an important operational consideration for sites adopting Policy Option 2, where flagged cases are intended to be confirmed by IPC chart review rather than acted upon directly, and is consistent with our stated position that trend-level monitoring (rather than case-level diagnostic certainty) is the appropriate use of this administrative data approach. Increases in trends would still suggest an overall increase in infection and trigger the necessary IPC steps to identify and reduce infection risk.

To move this policy forward, engagement between academic researchers, clinicians, and operational decision makers was necessary. Evidence around implementation of policy suggests that successful engagement and collaboration between academics and knowledge users/decision makers requires research that is relevant and has clear recommendations, as well as being aligned with strategic goals of the decision makers.^15^ This supports policy adoption and knowledge translation. Additionally, researchers should be familiar with the policy making process and the knowledge users, and ensure availability for communication and questions.^15,16^ In the current work, these conditions were met by involving members of the research team who would also be some of the knowledge users of the policy change as well as including an IPC research scientist who was able to bridge the connection between the academic team and operational decision makers. While replicating these relationships may not be practical for every type of policy implementation it is critical to consider how to optimize these factors when trying to enact policy change.^17^

Administrative data may offer an alternative to comprehensive IPC surveillance for CIED infections, or a hybrid option that can be used to supplement traditional surveillance. This can allow for the tracking of infection rates and careful foundational work has led to an evidence-informed policy decision change to use this for the province of Alberta. Future studies should endeavor to apply this work to other types of surgical infections, and our group is currently exploring using administrative data to track CIED infections nationally across Canada which will demonstrate widespread generalizability of this novel mechanism for IPC surveillance.

## Supporting information

10.1017/ash.2026.10874.sm001 Rennert-May et al. supplementary material 1 Rennert-May et al. supplementary material

10.1017/ash.2026.10874.sm002 Rennert-May et al. supplementary material 2 Rennert-May et al. supplementary material

## Supplementary material

The supplementary material for this article can be found at https://doi.org/10.1017/ash.2026.10874

## Figure 1. Long description

The table is titled ‘Cardiac Implantable Electronic Devices Surgical Site Infection Surveillance Demonstration Project Facility Surveillance Dashboard’. It has six rows and five columns. The columns are labeled ‘Implanting Facility’, ‘Procedures’, ‘SSI Rate’, ‘Days Since Last Infection Event Spark Line’, and ‘SSI Free Days’. The rows list different hospitals: St. Eligius Hospital, County General Hospital, Sacred Heart Hospital, Eastman Medical Center, Mobile Army Surgical Hospital, and Hope Memorial. Each row provides data on the number of procedures, SSI rate, days since the last infection event, and SSI-free days for each hospital. Notable trends include varying SSI rates and days since the last infection event across the hospitals.

Navigate back to Figure 1..
