# Supplementary material for: Enacting policy to use administrative data for surveillance of cardiac implantable electronic device infections in Alberta, Canada
Source: Antimicrob Steward Healthc Epidemiol. 2026 Oct 1;6(1):e307. doi: 10.1017/ash.2026.10874 (PMC13760398; doi:10.1017/ash.2026.10874)
Supplement: Supplementary file 1 [file S2732494X26108742sup002.pdf]

Supplemental Material 2

Briefing note

Title: Surveillance for Surgical Site Infections following Cardiac Implantable Electronic Devices

<u>For Information</u>

This document is to summarize the possibility of using administrative data to conduct surveillance on surgical site infections (SSIs) of cardiac implantable electronic devices (CIED) in Alberta. This document summarizes work done to date to validate administrative data and evaluate the epidemiology of CIED infections in Alberta. This work was presented at a meeting with relevant knowledge users, including members of the IPC Surveillance and Standards Team, to gain feedback on work done to date and the feasibility of using administrative data for provincial surveillance. This document also summarizes relevant discussions and topics from that meeting.

Executive Summary:

Tracking of surgical outcomes, such as post-surgical infection, in patients is an essential component of monitoring health system performance and ensuring delivery of high-quality care. One type of infection that is increasingly prevalent is surgical site infection (SSI) from cardiac implantable electronic devices (CIED). CIED SSIs have been identified as a priority within healthcare systems given their increasing frequency, substantial morbidity for patients, and significant economic burden to the healthcare system. Appropriate infection prevention and control (IPC) surveillance allows for recognition and targeted interventions when rates of infection increase beyond acceptable targets. However, comprehensive IPC surveillance programs are resource-intensive, costly, time-consuming, and unsustainable, particularly in many centers with limited funding for IPC surveillance programs. An attractive alternative is the use of already collected, health system administrative data to identify SSIs.

Relevant Alberta Health Services Foundational Strategies:

**Patient First Strategy**	**Our People Strategy**	**Information Management/Information Technology Strategy**	**Clinical Health Research, Innovation and Analytics Strategy**
		X	X

Background and Current Situation:

CIEDs are among the top 10 most common and expensive implantable medical device procedures in Canada. Accordingly, tracking post-surgical infections of CIEDs is paramount to monitoring health-system performance and ensuring high-quality care. The scope of CIED infections is broad with significant consequences, including impaired quality of life, increased mortality, significant morbidity, and lengthy hospitalization. CIED infections are also a large economic burden within the healthcare system. Currently, there are no national data or province-wide data for Alberta for CIED infections. Quality tracking of CIED infections is a priority, which can be done through IPC surveillance programs. However, comprehensive IPC surveillance programs are resource-intensive, costly, time-consuming, and unsustainable, particularly in many centers with limited funding for IPC surveillance programs.

We propose the use of administrative data to conduct province-wide CIED infection surveillance; specifically using the administrative data to monitor CIED infection trends at each hospital site where CIEDs are implanted.

Surveillance and Report Options Discussed:

Please see the tables below for the various options discussed for the use of administrative data and for what to include in a dashboard for surveillance reports.

Surveillance Options:

Option #	Use Options	Description<br>Description of what alternative entails	Pros	Cons
1	**Only use the admin data for CIED surveillance for entire Province**	This method would see the discontinuation of any traditional IPC committee and surveillance. Instead, only admin data would be used to monitor CIED infection trends across the province and sites.	• Excellent sensitivity and specificity in admin data<br>• Cost and resource effective – would reduce ICP workload<br>• Would be province wide	• Low positive predictive value<br>• False positives for CIED infection cases leads to concerns about accuracy.<br>• If trends change, no process set-up on how to investigate those changes
2	**Use the admin data to monitor trends and if there is a change in trends, this then triggers more intensive IPC surveillance**	This method would use the admin data to monitor CIED infection trends across the entire Province for each individual site. If the admin data shows a change in trends for a certain site, traditional IPC surveillance would be launched to investigate.	• More cost effective than traditional IPC<br>• IPC could verify cases when triggered<br>• All sites in the province would have some type of surveillance available<br>• This would utilize the admin data	• Resources required for IPC<br>• Process is needed to determine when a trigger is set, and what intensive IPC surveillance would entail.<br>• High false positive rate of admin data leads to concerns about accuracy

Option #	Use Options	Description<br>Description of what alternative entails	Pros	Cons
			effectively<br>• Admin data has high sensitivity and specificity	
3	**Do not use admin data and continue with only traditional IPC surveillance**	Keep the current CIED infection surveillance system in place and make no changes. IPC surveillance would continue at set intervals for certain sites.	• IPC surveillance is the 'gold standard'<br>• Committee and processes already exist at two sites	• Resource intensive<br>• Some sites may continue to not be able to conduct CIED infection surveillance due to resource constraints

Reporting Options

#	Reporting Options-	Pros	Cons	Who would have access?
1	**Tableau Dashboard vs. Manual IPC reports**	**Tableau:** Registered users can have access to almost just-in time data<br>• Graphics are visually appealing<br>• Graphics can be manipulated by the end-user to display only what they want<br>• Time savings overall by analysts<br><br>**Manual Reports:** Established reports by IPC for SSIs already exist.<br>• Only shared to relevant partners<br>• Does not require expertise in IT, SQL coding, Tableau development.<br>• Appropriate for outcomes with small numbers	**Tableau:** Requires identification of all important users requiring access.<br>• Permissions would need to be managed<br>• Risk of misinterpretation of figures, small case counts, if shared broadly (intentionally or accidentally).<br>• Requires ongoing management of extracts, SQL coding, tableau dashboard managements by analysts.<br><br>**Manual Reports:**<br>• Time and resource intensive<br>• Increased risk of errors<br>• Limitations in graphics<br>• Increased risk of misinterpretation of figures, small case counts by non-relevant partners (report shared on Internal AHS website)	**Tableau:** Registered users identified by relevant partners (e.g. IPC staff and physicians, cardiac/EP surgeons).<br><br>**Manual Reports:** IPC, members of the IPC provincial committees, local committees at sites where CIEDs performed. Reports emailed to relevant partners and posted on internal AHS website.
2	**Include both infection rate and**	• General consensus from meeting with	• Time since infection will represent when	Available generally as QI indicator.

#	Reporting Options-	Pros	Cons	Who would have access?
	**time since last infection (by site)**	knowledge users and collaborators was that these are both useful measures to include in a report	the infection was identified by healthcare providers, not necessarily when the infection initially presents<br>• Need to make sure looking at primary procedures and not revisions	
3	**Site-based reports**	• Valuable QI indicator	• Information could be sensitive (i.e. individual patients and operators may be identifiable at smaller sites)<br>• Need to maintain privacy	Specific sites would receive their rate/site-based report
4	**Include 1-yr follow-up period in report instead of 90-days**	• Infections beyond 90 days are not uncommon so this could make sure all infections are being caught	• Need to obtain data.<br>• Delay in data reporting because waiting for follow-up period to complete.<br>• Continuous updating of the data increases risk of errors.	Available generally as QI indicator

Engagement:

	**Engagement Status**	**What has been achieved to date?**	**What is planned?**
**External**	Engaged	IPC representatives have been introduced to using admin data for surveillance and have been invited to meetings held about this. Further, meetings have taken place with relevant individuals at rural hospitals to inform them of this project. Additionally, this project has a patient partner to provide further insight into the patient experience and how we can better serve patients.	See communications plan
**Clinicians**	Engaged	Cardiologists have been invited to meetings about using admin data for surveillance.	See communications plan

Communications Plan:

Recommendations for Future Engagement

To ensure patients are receiving the best possible care and efficacy of CIED surveillance, committees comprised of IPC professionals, operators, and other relevant knowledge users should be created at each hospital site within the province. These committees would meet regularly to review trends in the administrative data and review specific cases. In these committee meetings, the reports would be provided for everyone to review. While the committee will take time and resources at each hospital site, the pay-off will be valuable. Committee members will review the information in the reports, verify cases if needed, and create plans for reducing CIED infections if needed. Then, these members will disseminate the information to the wider, relevant hospital team (I.e., nurses, unit clerks, etc.). These formal meetings could make knowledge dissemination more efficient.

An extended discussion was had on feedback loops and who, exactly, should have access to the reports. There was consensus among the IPC teams that sharing reports, both formally and informally, was highly beneficial and proved to see high levels of engagement to key knowledge users receiving reports. The key knowledge users identified were:

- medical corridor directors
- site operations
- surgical leads
- unit managers
- physician leads of CIED device programs
- educators at device clinics
- individual cardiac surgeons/implanting cardiologists
- and infection control professionals.

These key knowledge users would receive slightly different information depending on which of these categories they fall under. For example, site chiefs would receive site specific rates in their report versions, and operations would receive only the overall rates in their report versions. The reports would otherwise be comprehensive for everyone unless falling under a category where more information would be received concerning them specifically. Sharing the reports with unit managers, directors, and lead physicians would be done with the understanding that these individuals would disseminate and share the information with the rest of their staff or direct them to the dashboard or internal AHS IPC website to help keep everyone informed and engaged. Generally, non-sensitive information, such as overall rates, would be available to the general AHS audience through the reports, with more sensitive information, if available, having

restricted access. Concerns were also identified about operator confidentiality if operator reports were generated. However, there are not any plans to collect individual operator data as this is not a current possibility with the administrative data.

Next Steps:

Our initial next steps are as follows:

1. Disseminate this document to the IPC Surveillance & Standards team
2. Meet with the IPC Surveillance & Standards team
3. Identify next actions for research and the IPC Surveillance & Standards team
