# Supplementary material for: Enacting policy to use administrative data for surveillance of cardiac implantable electronic device infections in Alberta, Canada
Source: Antimicrob Steward Healthc Epidemiol. 2026 Oct 1;6(1):e307. doi: 10.1017/ash.2026.10874 (PMC13760398; doi:10.1017/ash.2026.10874)
Supplement: Supplementary file 2 [file S2732494X26108742sup001.docx]

Supplemental Material 1

Post Workshop Follow up Questions:

1. Do you have any questions about the project or what was presented in the meeting?
2. Is there a benefit/need of surveillance for CIED infections?
3. Is there interest in doing provincial CIED SSI* surveillance?
4. What are concerns around using administrative data, and what are felt to be the benefits?
5. What information is important to include for reporting on CIED SSIs?
6. Do you have a preference on the format of the reports? If so, which format?
7. In your opinion, what are the pros and cons of each format?
8. Who should have access to the surveillance reports?
9. How can CIED SSI surveillance reports get to the operators/surgeons?
10. Is there other information that is necessary to help make a decision on the use of administrative data for CIED SSI surveillance? If so, is this something the research team can support?
11. What are the benefits to patients directly and indirectly?

*SSI: Surgical site infections
